# Where antibiotic time-outs work: department- and ward-stratified effects of a pharmacist-led anti-MRSA time-out

**DOI:** 10.1017/ash.2026.10778

**Published:** 2026-07-09

**Authors:** Risako Yamamoto, Yasuaki Tagashira, Do Dang An, Yui Enatsu, Maya Satake, Yoshibumi Aiso, Yoshiaki Gu

**Affiliations:** 1 Department of Pharmacy, Institute of Science Tokyo Hospital, Bunkyo, Tokyo, Japan; 2 Department of Infection Control, Institute of Science Tokyo Hospital, Bunkyo, Tokyo, Japan; 3 Institute of Science Tokyo Graduate School of Medical and Dental Sciences, Tokyo, Japan; 4 Center for Infectious Disease Education and Analysis, Institute of Science Tokyo, Tokyo, Japan

## Abstract

**Objective:**

To assess the impact of a pharmacist-led anti-methicillin-resistant *Staphylococcus aureus* (MRSA) agents’ time-out and identify clinical areas most likely to benefit.

**Design:**

Single-center before–after interrupted time-series study.

**Setting:**

An 813-bed tertiary hospital in Tokyo, Japan.

**Patients:**

Inpatients receiving intravenous anti-MRSA agents.

**Methods:**

Stewardship pharmacists alerted physicians to reassess intravenous anti-MRSA therapy at 72 hours after its start. Monthly days of therapy per 1,000 patient-days (DOT) were compared between October 2017 to September 2022 and October 2022 to September 2024 after stratification by ward and department. Acceptance rate and therapeutic drug monitoring (TDM) tests were also assessed.

**Results:**

Hospital-wide DOT showed an immediate non-significant decrease (−19.23; 95% confidence interval [CI] −59.17 to 20.72; *P* = .35) and no significant trend change (+0.82; 95% CI −1.18 to 2.82; *P* = .42). In emergency medicine, DOT decreased in critical care (slope change −20.3; 95% CI −36.25 to −4.28; *P* = .01) and general wards (−31.6; 95% CI −61.4 to −1.79; *P* = .04). In emergency medicine critical care, vancomycin use decreased (level change −406.1; 95% CI −801.3 to −10.9; *P* = .04) with a reduced trend (slope change −24.5; 95% CI −41.2 to −7.8; *P* < .001). Acceptance was higher in critical care than in general wards (77.1% [27/35] vs 33.6% [40/119]). TDM tests per 1,000 patient-days decreased (8.47 ± 2.39 to 6.55 ± 1.18; *P* < .001), with no increase in length of stay or in-hospital mortality.

**Conclusions:**

Targeting an implementation to areas most likely to benefit from it may improve antimicrobial stewardship when resources are limited. Complementary strategies may be needed if acceptance is poor.

## Introduction

Antimicrobial resistance is a major public health problem,^1^ and infections caused by antimicrobial-resistant organisms are associated with a longer hospital stay and higher mortality rate than those caused by antimicrobial-susceptible organisms. Appropriate antimicrobial use is vital to ensuring treatment efficacy and reducing unnecessary exposure, thereby decreasing adverse events and selective pressure on pathogens. Antimicrobial stewardship (AS) reduces antimicrobial use without increasing mortality and helps prevent the emergence of resistant organisms, including Clostridioides difficile.^2–5^

Guidelines recommend discontinuing antimicrobials after 72 hours absent clear evidence of infection requiring therapy.^6^ The Centers for Disease Control and Prevention (CDC) also encourage reassessing the need to continue antimicrobial therapy after the initial treatment period.^7,8^ However, this reassessment is often not performed even when additional diagnostic information becomes available.^8^

Antimicrobial time-out is an AS intervention that prompts reassessment of several parameters—the appropriateness of the diagnosis, choice of drug, dosage, administration route, and treatment duration at a predefined time point (generally 48–72 h) after its start. The pharmacist-led variant—in which a pharmacist alerts the prescriber to trigger reassessment—has been endorsed by the Infectious Diseases Society of America and the CDC as highly feasible.^8,9^ However, previous studies of the time-out approach have produced inconsistent findings.^10–12^ One study that implemented time-out by department found pharmacist-led time-out associated with reduced vancomycin use in the emergency and surgery departments but showed no significant change in other departments.^13^ This department-dependent response suggests that a time-out’s efficacy may vary by department and ward rather than producing uniform reductions across clinical areas.

Intravenous vancomycin, a glycopeptide antibiotic, is the main treatment for gram-positive infections, including those caused by methicillin-resistant Staphylococcus aureus (MRSA). The drug is also widely used as empirical therapy when a healthcare-associated infection (HAI), such as a catheter-related bloodstream infection or surgical site infection, is suspected; however, vancomycin choice or continuation has been deemed inappropriate in approximately 20%–70% of cases.^14–16^ Therefore, optimizing vancomycin and other anti-MRSA agents is an important AS objective.

Although ASPs are the cornerstone of efforts to protect society against antimicrobial-resistant pathogens, they intensively require human resources and face many constraints on their implementation. In Japan, pharmacists often lack time to dedicate to ASP-related activities,^17^ and in Latin America, a shortage of clinical pharmacists and a similar lack of time have been identified as key barriers.^18,19^ Designing interventions for maximum efficacy under conditions of resource scarcity—for example, by identifying which departments and wards should be prioritized—is therefore equally important. We aimed to evaluate the impact of a pharmacist-led anti-MRSA time-out stratified by department and ward and to identify the groups most likely to benefit.

## Methods

### Study design and setting

This before–after study was conducted at the Institute of Science Tokyo Hospital (ISTH, formerly Tokyo Medical and Dental University Hospital), an 813-bed tertiary care hospital in Tokyo, Japan. The preintervention period was October 1, 2017, to September 30, 2022, and the postintervention period was October 1, 2022 to September 30, 2024. The study was approved by the institutional review board of ISTH (M2024-035). Because this was a minimal-risk quality improvement activity without direct patient intervention, the requirement for informed consent was waived.

The antimicrobial stewardship team (AST) is operated by stewardship pharmacists with a 0.9 full-time equivalent (FTE) and an infectious diseases specialist with a 0.1 FTE. The main ASP activities include: (1) postprescription audit and feedback for broad-spectrum antimicrobials (carbapenems and piperacillin/tazobactam) and intravenous antimicrobials administered ≥14 days; (2) prospective audit and feedback (PAF) for intravenous fluoroquinolones; (3) a preauthorization notification system for anti-MRSA agents and antimicrobials with anti-Pseudomonas activity; (4) a restriction policy requiring approval for daptomycin, linezolid, and non-formulary antimicrobials; (5) a multidisciplinary antimicrobial time-out covering all intravenous antibiotics has been operational within ICU rounds since June 2018, conducted by pharmacists and limited to ICU patients, and infectious diseases consultation has been provided since June 2021.^20^

### Patients and intervention

Among all inpatients receiving an anti-MRSA agent (vancomycin, teicoplanin, linezolid, or daptomycin), those continuing therapy beyond 72 hours and meeting no exclusion criteria were included. The exclusion criteria were: 1) use of an anti-MRSA agent as first-line therapy (eg, MRSA bacteremia, methicillin-resistant coagulase-negative Staphylococcus bacteremia, or culture-negative cases judged appropriate for definitive therapy); 2) high epidemiologic risk (eg, known MRSA colonization, prior MRSA infection, or prolonged healthcare exposure). Anti-MRSA agents used solely as surgical prophylaxis were also excluded; this was completed within 72 hours unless a postoperative infection (eg, surgical site infection) was suspected and switched to treatment, continuing beyond 72 hours; 3) *β*-lactam allergy; and 4) the recommendations of an infectious diseases physician; 5) therapy completed before the 72-hour time-out. These criteria were defined based on prior research.^13^ When uncertain, pharmacists decided after discussion with infectious disease physicians.

For eligible cases, stewardship pharmacists contacted the prescriber by telephone at 72 hours to prompt reassessment of the continued need for anti-MRSA therapy; the time-out did not include specific recommendations to change therapy. If unreachable, pharmacists entered the same standardized EHR note based on available information. The standard operating procedure and example EHR notes are in Supplementary.

### Outcome

The primary outcome was monthly days of therapy per 1,000 patient-days (DOT) for all anti-MRSA agents combined and for each anti-MRSA agent, stratified by ward type (critical-care vs general) and department (emergency medicine, surgery, internal medicine, hematology, and pediatrics), generating ten analytical groups. Secondary outcomes were: (1) hospitalwide monthly DOT for all anti-MRSA agents combined and per agent; (2) duration of anti-MRSA therapy per case; (3) acceptance rate, the proportion of cases in which anti-MRSA therapy ended within three days of the time-out; (4) monthly therapeutic drug monitoring (TDM) tests per 1,000 patient-days; and (5) average monthly length of stay and in-hospital mortality. Pediatric data were analyzed separately because these patients consisted primarily of those with primary immunodeficiency or undergoing hematopoietic stem cell transplantation.

### Statistical analysis

Normality was assessed using the Shapiro–Wilk test. Welch’s *t* test was used for normally distributed continuous variables and the Mann–Whitney test for non-normally distributed variables; the χ^2^ test was used for categorical variables. To evaluate the intervention effect on monthly DOT, interrupted time-series analysis (ITSA) used 60 preintervention and 24 postintervention monthly time points to estimate level and slope changes. Stata version 18.0 (StataCorp, College Station, TX) was used for the ITSA analysis and R version 3.6.3 for other analyses.

## Results

### Antimicrobial time-out eligibility

**Figure 1.**
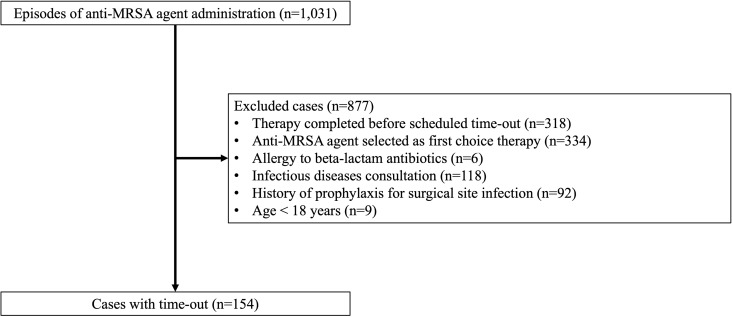
Flowchart of the intervention.

There were 3,937 inpatient anti-MRSA cases (2,906 preintervention; 1,031 postintervention). The patients’ median age (IQR) was 66 years (51–76) preintervention and 67 years (54–77) postintervention; 68% and 65% were male, respectively. Of the 1,031 postintervention cases, 877 (85%) met the exclusion criteria, and time-out was performed in 154 cases (15%) in Figure 1. Among the 154 eligible cases, the prescriber could not be contacted in 4 (2.6%), and in 15 (9.7%) the prescriber had proactively contacted the AST during other ASP activities. Reasons for exclusion by ward and department are in Table 1.

**Table 1.** Number of cases excluded from the time-out and the reasons for exclusion stratified by ward and clinical department Table 1 long description.

	All	Critical care ward					General ward				
		Emergency medicine	Surgery	Internal medicine	Hematology	Pediatrics	Emergency medicine	Surgery	Internal medicine	Hematology	Pediatrics
Exclusions	877	139	163	58	4	12	7	259	149	50	36
Therapy discontinued before scheduled time-out	318 (36.3 %)	82 (59.0 %)	45 (27.6 %)	31 (53.4 %)	0	7 (58.3 %)	2 (28.6 %)	74 (28.6 %)	56 (37.6 %)	14 (28.0 %)	7 (19.4 %)
Anti-MRSA agent used as first-line therapy	334 (38.1 %)	48 (34.5 %)	37 (22.7 %)	11 (19.0 %)	3 (75.0 %)	3 (25.0 %)	4 (57.1 %)	117 (45.2 %)	64 (43.0 %)	29 (58.0 %)	18 (50.0 %)
*β*-lactam allergy (anti-MRSA agent selected)	6 (0.7 %)	0	0	0	0	0	0	4 (1.5 %)	1 (0.7 %)	1 (2.0 %)	0
Infectious diseases consultations	118 (13.5 %)	9 (6.5%)	14 (8.6 %)	15 (25.9 %)	1 (25.0 %)	1 (8.3 %)	1 (14.3 %)	42 (16.2 %)	26 (17.4 %)	6 (12.0 %)	3 (8.3 %)
Surgical site infection prophylaxis	92 (10.5 %)	0	67 (41.1 %)	1 (1.7 %)	0	0	0	22 (8.5 %)	2 (1.3 %)	0	0
Age < 18 years	9 (1.0 %)	0	0	0	0	1 (8.3 %)	0	0	0	0	8 (22.2 %)

### Anti-MRSA agent use by ward and department

**Figure 2.**
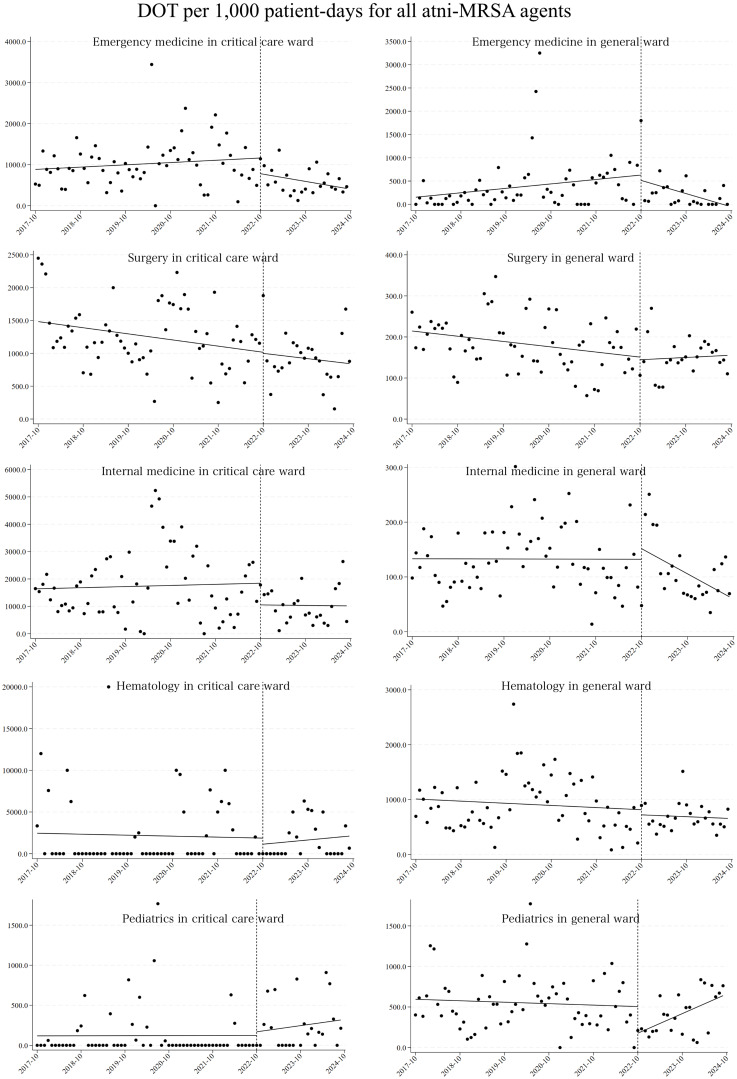
DOT trend for all anti-MRSA agents by ward and clinical department.

The trend in DOT by ward and department is shown in Figure 2. In the emergency department, DOT for all anti-MRSA agents combined declined significantly in both the critical-care ward (slope change −20.3; 95% CI −36.25 to −4.28; *P* = .01) and the general ward (slope change −31.6; 95% CI −61.4 to −1.79; *P* = .04); in the critical-care ward, vancomycin use decreased both immediately and significantly (level change −406.1; 95% CI −801.3 to −10.9; *P* = .04), with a continuing decrease (slope change −24.5; 95% CI −41.2 to −7.8; *P* < .001), while in the general ward only the trend indicated a significant decrease (slope change −34.4; 95% CI −62.9 to −5.87; *P* = .02). In the surgery department, overall anti-MRSA DOT showed no significant change, with a non-significant postintervention increase in both critical-care (slope change + 0.98; 95% CI −29.21 to 31.17; *P* = .95) and general wards (slope change + 1.52; 95% CI −1.46 to 4.49; *P* = .32); however, the duration of anti-MRSA therapy per case shortened significantly (−1.0 d; *P* < .05). In the internal medicine department, the critical-care ward showed an immediate and significant decrease in DOT (level change −793.3; 95% CI −1540.0 to −46.6; *P* = .04), with a non-significant trend, while the general ward showed a borderline decline (slope change −3.81; 95% CI −7.57 to −0.04; *P* = .05). In hematology, no statistically significant overall anti-MRSA DOT change was observed; in the general ward, vancomycin use decreased significantly (level change −14.9; 95% CI −24.53 to −5.18; *P* < .001) whereas teicoplanin use increased significantly (level change + 15.7; 95% CI 6.22 to 25.16; *P* < .001), suggesting agent substitution rather than overall reduction. In the pediatrics department, anti-MRSA DOT in the general ward showed an immediate significant decrease (level change −328.5; 95% CI −513.73 to −143.31; *P* < .001) followed by a significant rising trend (slope change + 21.6; 95% CI 11.06 to 32.19; *P* < .001), with vancomycin demonstrating an analogous pattern (level change −301.2; *P* < .001; slope change + 19.4; *P* < .001).

### Hospital-wide use of anti-MRSA agents

**Figure 3.**
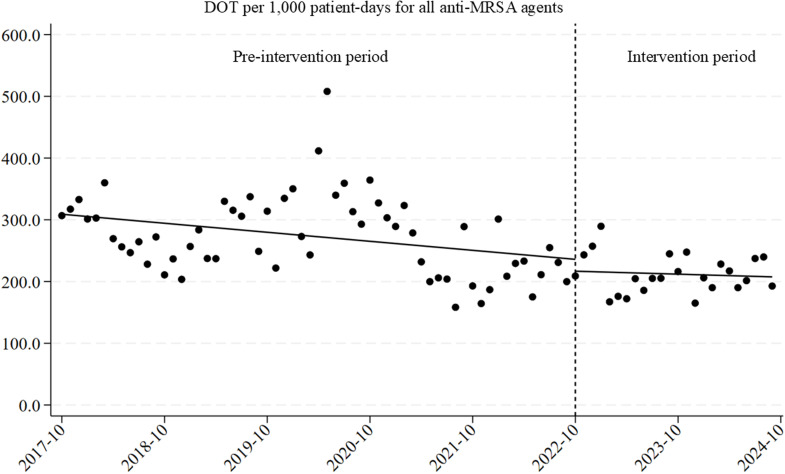
DOT trend for all anti-MRSA agents at the study center.

**Figure 4.**
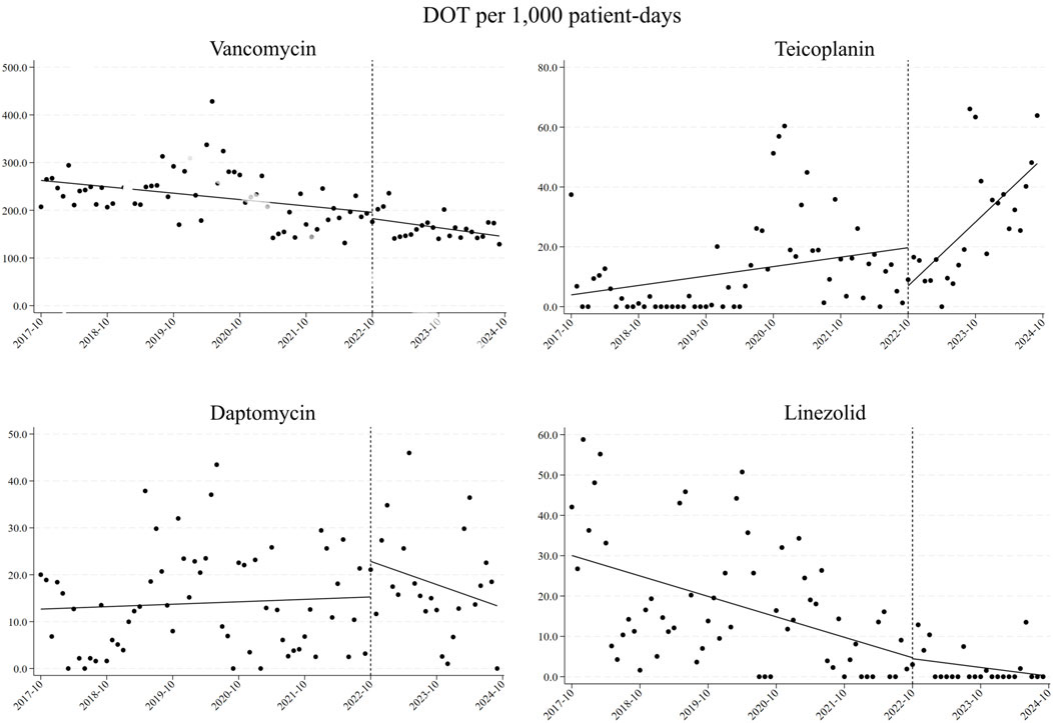
Figure 4 long description. DOT trend for each anti-MRSA agent at the study center.

Hospital-wide DOT showed a non-significant immediate decrease following the intervention (level change −19.23; 95% CI −59.17 to 20.72; *P* = .35) with no significant trend change (slope change + 0.82; 95% CI −1.18 to 2.82; *P* = .42) (Figure 3). Figure 4 shows the trend in DOT per agent.

### Other outcomes

**Figure 5.**
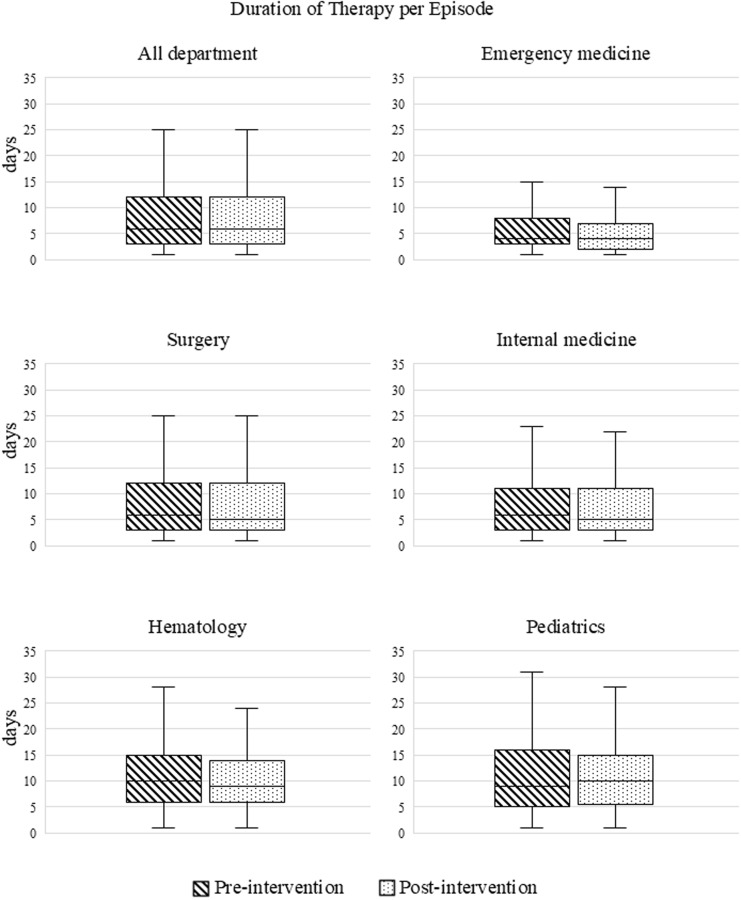
Comparison of the median duration of anti-MRSA therapy by clinical department before and after the intervention.

The overall acceptance rate was 43.5% (67/154). Acceptance was markedly higher in critical-care wards (77.1%, 27/35) than in general wards (33.6%, 40/119). Within critical-care wards, the rate by department was 76.0% (19/25) in emergency medicine, 100% (3/3) in surgery, and 66.7% (2/3) in internal medicine. Within general wards, acceptance was 81.0% (17/21) in surgery and 26.7% (4/15) in internal medicine; it was notably lower in hematology (24.2%, 16/66) and pediatrics (17.6%, 3/17). There were no eligible cases in the critical-care ward of the pediatric department or in the general ward of the emergency department (Table 2). Median anti-MRSA therapy duration per case by department before and after intervention is shown in Figure 5.

**Table 2.** Acceptance rate of the time-out stratified by ward and clinical department

Ward type and clinical department	Acceptance rate
All units	43.5% (67/154)
Critical care ward	
All	77.1% (27/35)
Emergency medicine	76.0% (19/25)
Surgery	100.0% (3/3)
Internal medicine	66.7% (2/3)
Hematology	75.0% (3/4)
Pediatrics	N.A.
General ward	
All	33.6% (40/119)
Emergency medicine	N.A.
Surgery	81.0% (17/21)
Internal medicine	26.7% (4/15)
Hematology	24.2% (16/66)
Pediatrics	17.6% (3/17)

Monthly TDM tests per 1,000 patient-days decreased significantly from 8.47 ± 2.39 preintervention to 6.55 ± 1.18 postintervention (*P* < .001), driven primarily by vancomycin (8.30 ± 2.38 to 6.06 ± 1.17; *P* < .001). Teicoplanin TDM tests increased significantly (0.17 ± 0.22 to 0.50 ± 0.34; *P* < .001). Average monthly length of stay (11.06 vs 10.60 d) and in-hospital mortality (2.25% vs 2.17%) showed no significant deterioration between periods.

## Discussion

In this study, a pharmacist-led anti-MRSA time-out produced few changes at the hospital-wide level; however, stratified analysis by department and ward demonstrated that it may have contributed to reducing the use of anti-MRSA agents in specific departments. In our data, the groups most likely to benefit were those with significant DOT reductions and high acceptance rates—most clearly the emergency department, where reductions were observed regardless of ward type. Prioritizing such departments and wards may therefore be useful when designing a more efficient ASP in settings lacking adequate human resources. At the same time, acceptance varied widely by department and ward, suggesting that where it was low, time-out alone may be insufficient and should be combined with other interventions, including PAF, department-specific consensus building, and systematic clinical support. Unlike PAF, in which the stewardship team evaluates the appropriateness of the prescription and provides culture-based feedback and specific recommendations to change therapy, the present time-out only prompted the attending physician to reassess the need for therapy; combining the two approaches may therefore be complementary.

Pharmacists play an important role in ASP by optimizing antimicrobial use, reducing medical costs, addressing antimicrobial resistance, and improving patient outcomes.^21–27^ Pharmacist-led interventions may shorten hospital stay and reduce broad-spectrum antimicrobial use.^28–30^ One pharmacist-led intervention for uncomplicated gram-negative bacteremia shortened therapy duration.^31^ A study dividing departments into internal medicine and surgery found pharmacist-led time-out may have decreased vancomycin use in the surgery department.^13^ By subdividing into departments and stratifying by ward type, we found that anti-MRSA use in the emergency department decreased significantly regardless of ward type, with high acceptance rates, suggesting that pharmacists’ recommendations more readily translate into reassessment here.

In Japan, patients admitted under the emergency department remain under its care rather than being transferred or discharged early; consequently, many were still admitted under emergency medicine at 72 hours and thus eligible for the time-out. The emergency department frequently manages sepsis and septic shock, and guidelines recommend selecting broad-spectrum antimicrobials including anti-MRSA agents as initial empirical therapy.^32^ Time-out at approximately 72 hours, when culture results and the clinical course become available, therefore provides a natural reassessment window to correct the initial empirical therapy. The high acceptance rate in emergency medicine is consistent with this interpretation: when the clinical rationale for discontinuation is clear and timely, physicians are more receptive to pharmacist recommendations. Notably, the absence of eligible cases in the general ward of the emergency department postintervention may reflect not only intervention-driven behavioral changes but also earlier discontinuation of anti-MRSA agents before the 72-hour threshold. Because our study did not capture cases discontinued before the time-out, future studies should examine the proportion of treatment courses discontinued within 72 hours.

In the surgery department, high acceptance rates coexisted with a non-significant increase in overall DOT and no reduction in DOT trend, yet the duration of therapy per case shortened significantly. Because cases with anti-MRSA agents as clear first-line therapy were excluded, it is likely that the duration of unnecessary cases was shortened, while the number of cases requiring anti-MRSA therapy increased during the same period. Interpreting DOT for surgery therefore requires concurrent tracking of microbiological background—including MRSA-related infection incidence, surgical site infection epidemiology, and blood culture positivity rates—which we did not capture.

In internal medicine, DOT showed a decreasing tendency, but the acceptance rate in general wards was low (26.7%), and the trend change was unclear. Acceptance heterogeneity across departments likely reflected differences in case-mix complexity, physician decision-making culture, and prior familiarity with stewardship recommendations. In areas with a low acceptance rate, more structured interventions beyond a telephone alert may be needed, such as feedback in departmental conferences, standardizing order sets and treatment templates, and collaborating more closely with infectious diseases specialists.^20^

In hematology, vancomycin use decreased, while teicoplanin use increased in the general ward, suggesting agent substitution rather than overall reduction. In immunocompromised and transplant populations, the threshold for continuing therapy may be higher due to disease severity and immunosuppressive status, and agent selection is more likely to reflect changes in renal function or pharmacokinetic considerations. Similarly, in the pediatrics department, unique factors in this population (centralized management of primary immunodeficiency and transplant cases) strongly influence prescribing patterns. Although some reports have demonstrated the efficacy of time-out in cancer centers,^33^ our findings suggest anti-MRSA use is less likely to decrease in hematology and pediatrics through standard time-out alone. Disease-specific, multidisciplinary strategies may be required in these settings.

The higher acceptance rate in critical-care wards across departments (77.1% vs 33.6% in general wards) may reflect a preexisting culture of daily antimicrobial reassessment, the influence of multidisciplinary rounds,^20^ and clearer clinical end points that facilitate de-escalation decisions. However, compared with the all-antibiotic time-out operated within ICU rounds, our time-out differed in two ways: (i) it focused on anti-MRSA agents and was delivered by pharmacists independently of rounds at 72 hours, and (ii) it was extended hospital-wide beyond the ICU. Critically, the emergency department—where the largest reductions were seen—was in a separate critical-care ward not covered by the ICU program, so the hospital-wide expansion explains the significant decreases in previously uncovered critical-care areas. Even where no clear change in overall anti-MRSA use is observed, a time-out may still reduce unnecessary antimicrobial exposure as one component of a multifaceted stewardship approach.

The significant reduction in TDM tests per 1,000 patient-days is a meaningful ancillary benefit. Although TDM helps reduce adverse events such as nephrotoxicity,^34^ unnecessarily prolonged anti-MRSA therapy generates avoidable TDM burden for pharmacists and laboratory staff.

Time-out has improved prescription appropriateness, shortened therapy, and reduced use of specific agents, but its effects on total antimicrobial consumption and patient outcomes are inconsistent.^11,12,35–37^ Some studies have found no significant reduction in antimicrobial use,^10^ suggesting time-out is not universally effective alone but more effective within a multifaceted ASP.^11^ In our study, time-out covered only 15% of anti-MRSA cases, and overall DOT changes were modest; the effects of other ASP activities and behavioral changes such as increased early discontinuation should therefore also be considered. Accumulating evidence on which methods suit which populations may help conduct ASP more effectively within limited resources.

Several limitations must be acknowledged. First, this was a single-center study, limiting generalizability. Second, only 15% of cases met time-out criteria, and DOT changes may have been confounded by concurrent ASP activities or system changes. Third, the incidence of infections requiring anti-MRSA agents was not adequately accounted for. Fourth, safety outcomes such as nephrotoxicity, retreatment, readmission, and microbiological outcomes were not sufficiently assessed. Fifth, the pharmacist FTE dedicated to the time-out was not evaluated. Finally, all implementing pharmacists were female; physicians’ acceptance may be influenced by pharmacist gender,^38^ though institutional culture, expertise, and relationships can exert similar influence. In addition, infectious diseases consultation became available in June 2021, before the intervention. Consultations were not provided for 118 of 1,031 cases (11.4%), and we could not fully disentangle their effect; ID consultation may have prompted some early discontinuation independently of the time-out. Future studies should examine the implementation process using qualitative or mixed-methods approaches to elucidate the contextual factors driving differential acceptance.

## Conclusion

Pharmacist-led anti-MRSA time-out may contribute to reducing the use of anti-MRSA agents in specific departments—particularly emergency medicine—without worsening in-hospital outcomes. Implementation focusing on areas most likely to benefit, identified through stratified analysis by department and ward, may provide a feasible approach to curbing overuse even in settings with limited time and resources. In areas where acceptance is low or the patient population is clinically complex, time-out alone is likely insufficient; complementary strategies including PAF, department-specific education, and closer infectious diseases specialist involvement should be considered.

## Supporting information

10.1017/ash.2026.10778.sm001 Yamamoto et al. supplementary material Yamamoto et al. supplementary material

## Data Availability

The data sets used and/or analyzed during the current study are available from the corresponding author on reasonable request.

## Supplementary material

The supplementary material for this article can be found at https://doi.org/10.1017/ash.2026.10778.

## Table 1. Long description

The table presents data on the number of cases excluded from the time-out and the reasons for exclusion, stratified by ward and clinical department. It has 10 rows and 11 columns. The columns are labeled as All, Critical care ward, and General ward, with sub-columns for Emergency medicine, Surgery, Internal medicine, Hematology, and Pediatrics. The rows are labeled as Exclusions, Therapy discontinued before scheduled time-out, Anti-MRSA agent used as first-line therapy, Beta-lactam allergy (anti-MRSA agent selected), Infectious diseases consultations, Surgical site infection prophylaxis, and Age under 18 years. Each cell contains numerical values representing the count of cases for each category. Notable trends include high exclusion rates in Emergency medicine and Surgery departments, and a significant number of therapy discontinuations in the Critical care ward.

Navigate back to Table 1..

## Figure 4. Long description

Four scatter plots depict the DOT per 1,000 patient-days for Vancomycin, Teicoplanin, Daptomycin, and Linezolid over time. Panel A: The scatter plot for Vancomycin shows the y-axis labeled DOT per 1,000 patient-days ranging from 0 to 500 and the x-axis labeled with dates from October 2017 to October 2024. The data points show a general decreasing trend with a slight increase after the intervention line. Panel B: The scatter plot for Teicoplanin shows the y-axis labeled DOT per 1,000 patient-days ranging from 0 to 80 and the x-axis labeled with dates from October 2017 to October 2024. The data points show an increasing trend, especially after the intervention line. Panel C: The scatter plot for Daptomycin shows the y-axis labeled DOT per 1,000 patient-days ranging from 0 to 50 and the x-axis labeled with dates from October 2017 to October 2024. The data points show a slight increasing trend before the intervention line and a decreasing trend after. Panel D: The scatter plot for Linezolid shows the y-axis labeled DOT per 1,000 patient-days ranging from 0 to 60 and the x-axis labeled with dates from October 2017 to October 2024. The data points show a decreasing trend over time, with a notable drop after the intervention line.

Navigate back to Figure 4..

## References

[1] GBD 2021 Antimicrobial Resistance Collaborators. 2024; Antimicrobial resistance collaborators. Global burden of bacterial antimicrobial resistance 1990-2021: a systematic analysis with forecasts to 2050. Lancet 404:1199–1226. 39299261 10.1016/S0140-6736(24)01867-1 PMC11718157

[2] Davey P , Marwick CA , Scott CL , et al. Interventions to improve antibiotic prescribing practices for hospital inpatients. Cochrane Database Syst Rev 2017;2:CD003543. 28178770 10.1002/14651858.CD003543.pub4 PMC6464541

[3] Baur D , Gladstone BP , Burkert F , et al. Effect of antibiotic stewardship on the incidence of infection and colonization with antibiotic-resistant bacteria and Clostridium difficile infection: a systematic review and meta-analysis. Lancet Infect Dis 2017;17:990–1001. 28629876 10.1016/S1473-3099(17)30325-0

[4] Malani AN , Richards PG , Kapila S , Otto MH , Czerwinski J , Singal B. Clinical and economic outcomes from a community hospital’s antimicrobial stewardship program. Am J Infect Control 2013;41:145–148. 22579261 10.1016/j.ajic.2012.02.021

[5] Tamma PD , Miller MA , Dullabh P , et al. Association of a safety program for improving antibiotic use with antibiotic use and hospital-onset clostridioides difficile infection rates among US hospitals. JAMA Netw Open 2021; 4:e210235. 33635327 10.1001/jamanetworkopen.2021.0235 PMC7910818

[6] Morgan DJ , Croft LD , Deloney V , et al. Choosing wisely in healthcare epidemiology and antimicrobial stewardship. Infect Control Hosp Epidemiol 2016;37:755–760. 27019058 10.1017/ice.2016.61 PMC6490173

[7] Pollack LA , Srinivasan A. Core elements of hospital antibiotic stewardship programs from the centers for disease control and prevention. Clin Infect Dis 2014;59(Suppl 3):S97–100. 25261548 10.1093/cid/ciu542 PMC6521960

[8] Centers for Disease Control and Prevention. Core Elements of Hospital Antibiotic Stewardship Programs. Atlanta (GA): US Department of Health and Human Services, CDC. 2019. https://www.cdc.gov/antibiotic-use/media/pdfs/hospital-core-elements-508.pdf. Accessed February 8, 2026.

[9] Barlam TF , Cosgrove SE , Abbo LM , et al. Implementing an antibiotic stewardship program: guidelines by the Infectious Diseases Society of America and the Society for Healthcare Epidemiology of America. Clin Infect Dis 2016;62:e51–77. 27080992 10.1093/cid/ciw118 PMC5006285

[10] Thom KA , Tamma PD , Harris AD , et al. Impact of a prescriber-driven antibiotic time-out on antibiotic use in hospitalized patients. Clin Infect Dis 2019;68:1581–1584. 30517592 10.1093/cid/ciy852

[11] Van Schooneveld TC , Rupp ME , Cavaleiri RJ , Lyden E , Rolek K. Cluster randomized trial of an antibiotic time-out led by a team-based pharmacist. Infect Control Hosp Epidemiol 2020;41:1266–1271. 32814610 10.1017/ice.2020.347

[12] Taylor AP , Coe K , Stevenson K , Wardlow L , Boghdadly ZE , Reed E. Clinical impact of an antibiotic time out initiative at an academic medical center. Hosp Pharm 2021;56:343–346. 34381272 10.1177/0018578719901274 PMC8326851

[13] Hasegawa S , Tagashira Y , Murakami S , et al. Antimicrobial time-out for vancomycin by infectious disease physicians versus clinical pharmacists: a before-after crossover trial. Open Forum Infect Dis 2021; 8:ofab125. 34189155 10.1093/ofid/ofab125 PMC8232390

[14] Junior MS , Correa L , Marra AR , Camargo LF , Pereira CA. Analysis of vancomycin use and associated risk factors in a university teaching hospital: a prospective cohort study. BMC Infect Dis 2007 7: 88. 17678541 10.1186/1471-2334-7-88 PMC2014772

[15] Kim NH , Koo HL , Choe PG , et al. Inappropriate continued empirical vancomycin use in a hospital with a high prevalence of meticillin-resistant Staphylococcus aureus. Antimicrob Agents Chemother 2015;59:811–817. 25403664 10.1128/AAC.04523-14 PMC4335878

[16] Snyder GM , Patel PR , Kallen AJ , Strom JA , Tucker JK , D’Agata EM. Antimicrobial use in outpatient hemodialysis units. Infect Control Hosp Epidemiol 2013;34:349–357. 23466906 10.1086/669869

[17] Maeda M , Muraki Y , Kosaka T , et al. Essential human resources for antimicrobial stewardship teams in Japan: estimates from a nationwide survey conducted by the Japanese Society of Chemotherapy. J Infect Chemother 2019;25:653–656. 31182329 10.1016/j.jiac.2019.05.012

[18] Fabre V , Cosgrove SE , Secaira C , et al. Antimicrobial stewardship in Latin America: past, present, and future. Antimicrob Steward Healthc Epidemiol 2022;2:e68. 36483374 10.1017/ash.2022.47 PMC9726506

[19] Fabre V , Secaira C , Cosgrove SE , et al. Deep dive into gaps and barriers to implementation of antimicrobial stewardship programs in hospitals in Latin America. Clin Infect Dis 2023 77(Suppl 1):S53–S61. 37406044 10.1093/cid/ciad184 PMC10321692

[20] Mishima Y , Nawa N , Asada M , et al. Impact of antibiotic time-outs in multidisciplinary ICU rounds for antimicrobial stewardship program on patient survival: a controlled before-and-after study. Crit Care Explor 2023;5:e0837. 36699244 10.1097/CCE.0000000000000837 PMC9829256

[21] Wickens HJ , Farrell S , Ashiru-Oredope DA , Jacklin A , Holmes A ; Antimicrobial stewardship group of department of health advisory committee on antimicrobial resistance and health care associated infections (ASG-ARHAI). The increasing role of pharmacists in antimicrobial stewardship in English hospitals. J Antimicrob Chemother 2013;68:2675–2681. 23825383 10.1093/jac/dkt241

[22] Cappelletty D , Jacobs D. Evaluating the impact of a pharmacist’s absence from an antimicrobial stewardship team. Am J Health Syst Pharm 2013;70:1065–1069. 23719885 10.2146/ajhp120482

[23] Molloy L , McGrath E , Thomas R , Kaye KS , Rybak MJ. Acceptance of Pharmacist-Driven Antimicrobial Stewardship Recommendations With Differing Levels of Physician Involvement in a Children’s Hospital. Clin Pediatr (Phila). 2017;56:744–751. 27872355 10.1177/0009922816678598

[24] Waters CD. Pharmacist-driven antimicrobial stewardship program in an institution Without infectious diseases physician support. Am J Health Syst Pharm 2015;72:466–468. 25736941 10.2146/ajhp140381

[25] Mas-Morey P , Ballesteros-Fernández A , Sanmartin-Mestre E , Valle M. Impact of clinical pharmacist intervention on antimicrobial use in a small 164-bed hospital. Eur J Hosp Pharm 2018;25:e46–e51. 31157066 10.1136/ejhpharm-2017-001307 PMC6457161

[26] Bartlett JM , Siola PL. Implementation and first-year results of an antimicrobial stewardship program at a community hospital. Am J Health Syst Pharm 2014;71:943–949. 24830998 10.2146/ajhp130602

[27] Sadyrbaeva-Dolgova S , Aznarte-Padial P , Jimenez-Morales A , Expósito-Ruiz M , Calleja-Hernández MÁ. , Hidalgo-Tenorio C. Pharmacist recommendations for carbapenem de-escalation in urinary tract infection within an antimicrobial stewardship program. J Infect Public Health 2020;13:558–563. 31685404 10.1016/j.jiph.2019.09.014

[28] Sawada K , Inose R , Goto R , Nakatani T , Kono S , Muraki Y. Effectiveness of noncertified pharmacist-led antimicrobial stewardship programs in a medium-sized hospital without an infectious disease specialist: a retrospective pre-post study. Open Forum Infect Dis 2023 10:ofad116. 36949877 10.1093/ofid/ofad116 PMC10026541

[29] Ohashi K , Matsuoka T , Shinoda Y , et al. Clinical outcome of pharmacist-led prospective audit with intervention and feedback after expansion from patients using specific antibiotics to those using whole injectable antibiotics. Eur J Clin Microbiol Infect Dis 2019;38:593–600. 30680565 10.1007/s10096-018-03465-z

[30] Okada N , Azuma M , Tsujinaka K , et al. Clinical impact of a pharmacist-driven prospective audit with intervention and feedback on the treatment of patients with bloodstream infection. Antibiotics (Basel) 2022;11:1144. 36139925 10.3390/antibiotics11091144 PMC9495130

[31] Fukuda T , Tanuma K , Iio S , Saito J , Komura M , Yamatani A. Impact of a pharmacist-led antimicrobial stewardship program on the number of days of antimicrobial therapy for uncomplicated gram-negative bacteremia in a community hospital. Cureus 2021;13:e14635. 34046272 10.7759/cureus.14635 PMC8140741

[32] Guarino M , Perna B , Cesaro AE , et al. Update on sepsis and septic shock in adult patients: management in the emergency department. J Clin Med 2023; 12:3188. 37176628 10.3390/jcm12093188 PMC10179263

[33] Tverdek FP , Aitken SL , Mulanovich VE , et al. Implementation of an automated antibiotic time-out at a comprehensive cancer center. Open Forum Infect Dis 2024;11:ofae235. 38798895 10.1093/ofid/ofae235 PMC11127483

[34] Schuts EC , Hulscher MEJL , Mouton JW , et al. Current evidence on hospital antimicrobial stewardship objectives: a systematic review and meta-analysis. Lancet Infect Dis 2016;16:847–856. 26947617 10.1016/S1473-3099(16)00065-7

[35] Graber CJ , Jones MM , Glassman PA , et al. Taking an antibiotic time-out: utilization and Usability of a self-stewardship time-out program for renewal of vancomycin and piperacillin-tazobactam. Hosp Pharm 2015;50:1011–1024. 27621509 10.1310/hpj5011-1011 PMC4750836

[36] Adams SM , Ngo L , Morphew T , Babbitt CJ. Does an antimicrobial time-out impact the duration of therapy of antimicrobials in the PICU? Pediatr crit care med. 2019;20:560–567. 10.1097/PCC.0000000000001925 31166288

[37] Salehi M , Arabi M , Khalili H , et al. Impact of an antimicrobial time-out program on antimicrobial consumption rate in hospitalized patients: a quasi-experimental study on the national antimicrobial stewardship program in Iran: Iranian antimicrobial stewardship program. J Pharm Health Care Sci 2025;11:41. 40390037 10.1186/s40780-025-00451-4 PMC12090655

[38] Vaughn VM , Giesler DL , Mashrah D , et al. Pharmacist gender and physician acceptance of antibiotic stewardship recommendations: An analysis of the reducing overuse of antibiotics at discharge home intervention. Infect Control Hosp Epidemiol 2023;44:570–577. 35670587 10.1017/ice.2022.136 PMC10754057

