# Supplementary material for: Where antibiotic time-outs work: department- and ward-stratified effects of a pharmacist-led anti-MRSA time-out
Source: Antimicrob Steward Healthc Epidemiol. 2026 Jul 9;6(1):e208. doi: 10.1017/ash.2026.10778 (PMC13352333; doi:10.1017/ash.2026.10778)
Supplement: Yamamoto et al. supplementary material [file S2732494X26107785sup001.docx]

**Supplementary article.**

**1, The standard operating procedure**

**1. Purpose**

Anti-MRSA agents are among the broad-spectrum antimicrobials, and their inappropriate use has frequently been reported; antimicrobial stewardship is therefore required.　At Tokyo Metropolitan Tama Medical Center, a time-out for vancomycin conducted jointly by pharmacists and infectious-disease physicians has been reported, and the effect of the pharmacist-led time-out has been demonstrated.　Linezolid and daptomycin are already subject to pre-authorization; by adding a pharmacist-led time-out for anti-MRSA agents, we aim to further promote their appropriate use.

**2. Daily schedule**

Step 1. By 9:00 on weekdays, ASP pharmacist identifies patients who have been on an anti-MRSA agent for 3 days and enters the following information into “All Cases.xls” in the shared “Time-out” folder of the electronic health record (EHR): patient ID, patient name, ward, clinical department, the date of anti-MRSA drug start, the date of time-out, days of therapy (automatically calculated), and presence or absence of an initial trough level.

Step 2. By 10:00, ASP pharmacist reviews the cases to be excluded. If it is unclear whether exclusion is appropriate, the pharmacist confirms with the infectious-disease physician at the 10:00 microround.

Exclusion criteria:

– β-lactam allergy together with a confirmed Gram-positive coccus (GPC) infection

– A confirmed infection for which vancomycin is the first-line agent

– Prophylactic antimicrobial use

– Completion within 72 hours

– The patient is co-managed by, and continuation is recommended by, the Department of Infectious Diseases

Step 3. As appropriate, telephone the attending team and document in the EHR.

– Contact up to three members of the patient’s attending team.

– Document in the chart.

Telephone script: “This is [name], a pharmacist of the Antimicrobial Stewardship Team, Department of Infection Control. Regarding your patient, [name], more than 72 hours have passed since the anti-MRSA agent was started. Please review the culture results and consider whether to continue therapy. If there is no record of a positive blood culture in the chart, it is currently negative; a blood culture that is negative at 72 hours remains negative in 98% of cases. If you continue therapy, please measure trough levels 1–2 times per week; therapeutic drug monitoring (TDM) is performed by the Pharmacy Department. If it is difficult to decide on discontinuation or change, please consider consulting the Department of Infectious Diseases. Thank you very much.”

**3. Points to note**

– Do not answer questions regarding the necessity of continued administration.

– Respond using the response examples below.

– If you are unsure, contact the on-call staff of the Department of Infection Control.

– If the conversation moves toward an infectious-disease consultation, convey the name of that day’s on-call physician for infectious-disease consultations.

Response examples (responses to questions from the attending physician):

**Q1. At this point, is it all right to stop vancomycin?**

A1. This call is part of the time-out, an antimicrobial stewardship program. We are sorry, but we are not reviewing detailed information such as culture results on our end, so we cannot make that judgment. As a rule, if there is no positive entry in the chart, the blood culture is negative at this point. If you would also like to refer to other culture results, we would be grateful if you could contact extension [phone number].

**Q2. I am thinking of requesting an infectious-disease consultation; how should I do that?**

A2. Thank you for your question. We can either convey this to the Department of Infectious Diseases on your behalf, or you may contact [name], today’s on-call physician for infectious-disease consultations. Which would you prefer?

**Q3. The time-out is a nuisance for us when we are busy.**

A3. We apologize for calling while you are busy. However, because vancomycin is nephrotoxic and requires periodic monitoring of blood concentrations, we are working to promote its appropriate use. We appreciate your cooperation.

**Q4. What exactly does the antimicrobial stewardship program involve? Does it have any benefit?**

A4. The antimicrobial stewardship program aims to ensure that antimicrobials are used against bacterial infections with the appropriate agent, for the appropriate duration, and without adverse effects. It is important to use the necessary antimicrobials properly when they are needed and to discontinue them early when they are unnecessary. Research has shown that adverse effects of antimicrobials are more common than generally assumed. Worldwide, including in Asia, antimicrobial stewardship programs are known to safely reduce unnecessary antimicrobial use and improve patient outcomes.

**Q5. I am busy right now—could you keep it brief?**

A5. We apologize for interrupting while you are busy. When you have time, please consider whether vancomycin should be continued. The information is also posted in the chart, so we would be grateful if you could review it.

**Q6. It seems out of place for you to be the one telling me this; I do not accept it.**

A6. Thank you for your feedback. We pharmacists have the goal of ensuring that medications are used safely. Vancomycin, as an agent with anti-MRSA activity, is important for in-hospital infections, but it can cause renal impairment, and safe use requires periodic measurement of vancomycin concentrations. As one of the duties of us ward pharmacists, we carry out this program together with the Department of Infection Control and the Department of Infectious Diseases.

**Q7. But this patient currently has febrile neutropenia (FN). / But this is a super-high-risk patient.**

A7. The time-out is simply an initiative to notify you that 72 hours have elapsed since culture collection. Cultures often become positive within 72 hours; however, when blood-cell counts are low, cultures may become positive after about 5 days, so continuation is acceptable at your discretion. Regarding trough levels, the Pharmacy Department can assist with simulation. We are sorry to have interrupted you.

**2, Example EHR notes**

The following note is posted in the patient’s chart and on the bulletin board; identical wording is used in both.

Anti-MRSA drug was started on [Month/Day], and more than 72 hours have now elapsed. Please review the culture results and consider whether to continue, discontinue, or change therapy. If therapy is continued, please measure trough levels 1–2 times per week; therapeutic drug monitoring (TDM) is performed by the Pharmacy Department. If the situation is difficult to judge, please consider consulting the Department of Infectious Diseases.

Department of Infection Control, Antimicrobial Stewardship Team, [name]
